# Effects of organic zinc on production performance, meat quality, apparent nutrient digestibility and gut microbiota of broilers fed low-protein diets

**DOI:** 10.1038/s41598-023-37867-7

**Published:** 2023-07-04

**Authors:** Liping Dong, Yumei Li, Yonghong Zhang, Yan Zhang, Jing Ren, Jinlei Zheng, Jizhe Diao, Hongyu Ni, Yijing Yin, Ruihong Sun, Fangfang Liang, Peng Li, Changhai Zhou, Yuwei Yang

**Affiliations:** 1 College of Animal Sciences, The Jilin Provincial Key Laboratory of Livestock and Poultry Feed and Feeding in the Northeastern Frigid Area, Jilin University, Changchun, 130062 China; 2 College of Animal Science and Technology, Jilin Agriculture Science and Technology University, Jilin, 132109 China; 3 International Trading (Shanghai) Co., Ltd., Shanghai, 200080 China

**Keywords:** Biochemistry, Microbiology, Zoology

## Abstract

The high cost of feed and nitrogen pollution caused by high-protein diets have become major challenges restricting sustainable development in China's animal husbandry sector. Properly reducing protein levels and improving protein utilization in feed are effective approaches to solving this problem. To determine the optimal dose of methionine hydroxyl analogue chelated zinc (MHA-Zn) in broiler diets with a 1.5% reduction in crude protein (CP), a total of 216 1-day-old broilers were randomly assigned into 4 groups (each group consisted of 3 replications with 18 broilers per replicate), and growth and development indexes were assessed after 42 days. The broilers in control group were fed a basic diet, whereas those in the three test groups were fed diets with a 1.5% reduction in CP. The results showed no significant difference in the edible parts of broilers between low-protein (LP) diet group (90 mg/kg MHA-Zn) and normal diet group (*p* > 0.05), and adding 90 mg/kg MHA-Zn to LP diet significantly improved ileum morphology and apparent total tract digestibility (ATTD) of nutrient (*p* < 0.01; *p* < 0.05). A 16S rRNA sequencing analysis indicated that supplementing the LP diet with 90 mg/kg MHA-Zn was adequate for production performance of broilers and promoted beneficial bacteria in the cecum (*Lactobacillus*, *Butyricoccus*, *Oscillospira*, etc.) (*p* < 0.01). In summary, adding an optimal dose of organic zinc (90 mg/kg MHA-Zn) in low protein diets led to enhanced production performance of broilers and optimized cecum microbiota. Additionally, the reduction of crude protein consumption in broiler production proved to be a cost-effective measure, while also mitigated nitrogen pollutant emissions in the environment.

## Introduction

Poultry meat production is beneficial to resource conservation and environment, and the consumption of poultry meat is better for human health than that of red meat. Global consumption of poultry meat has increased per capita since 1960, and this trend is expected to continue until 2030^1,2^. As a result, it is vital for the poultry industry to reduce feed costs while improving poultry production and meat quality through various means. For example, as dietary protein sources are often an expensive component of poultry diets, optimizing a major of the protein in diets can reduce feed costs and environmental pollution, such as nitrogen excretion^3^. Nitrogen is mainly excreted as urea in urine (50%), while a small amount is excreted in feces (20%) during protein metabolism in animals^4^. Hence, livestock and poultry breeding wastewater usually have high concentrations of nitrogen and phosphorous, and its discharge may pose a great threat to the local water environment. Moreover, the water intake of animals is proportional to protein content of feed. Various studies have already illustrated that every 10 g/kg reduction in the dietary CP of fattening pigs can decrease ammonia (N_2_O) emission in feces and urine by 8% to 10%^4,5^, respectively, and the levels of serum urea nitrogen (SUN) and blood urea nitrogen (BUN) were also decreased significantly in animals fed the LP diet compared to those fed a standard diet^6,7^. It is not advisable to reduce CP content blindly in poultry diets. Several studies have demonstrated that a reduction in CP content of more than 3% in poultry diets will lead to decreased performance, carcass, and production traits of broilers^8–10^. Selection of appropriate Zn sources in diets for broilers is considered an appropriate alternative strategy and is the key method used to ensure that poultry will not be affected by reductions in protein level in the process of poultry feeding.

It is known that Zn is an essential trace mineral in poultry nutrition that participates in numerous metabolic pathways and biological functions, such as growth, feather and skeletal development and reproduction. The requirement of Zn for broilers is 40 mg/kg diet according to the NRC (1994), but supplementing Zn above NRC recommendations is a common practice in most countries and may be beneficial for the production of animals^11^. Conventional practices commonly add two inorganic Zn sources (Zn sulfate and Zn oxides) to ensure nutritional requirements are met in livestock feed^12^. The development of organic Zn sources as feed additives is an increasing trend in feed industry. Evidence from research has indicated that inorganic Zn is relatively inexpensive, but it has multiple disadvantages, including highly hydrophobic properties and excretion half-lives that last for decades compared with organic Zn. The low utilization ratio of inorganic Zn indicates that it is a waste of resources and causes environmental pollution^13^. Conversely, due to its ability to be absorbed more easily and its greater chemical and physical stability, organic Zn sources are recommended widely in animals, such as Zn gluconate and MHA-Zn^14^, and lower concentrations of these sources can meet feeding requirements^15^. Several studies have documented that adding MHA-Zn to feed has a beneficial effect on the quality of poultry meat, including drip loss reduction^16^. Moreover, methionine chelates can directly cross the intestinal cell membrane and be metabolized without any prior digestion due to being chelated with amino acids^17^, so this study was conducted to further investigate the effects of MHA-Zn.

The intestinal microbial flora plays an important role in animal health and has attracted increasing attention in recent years. Researchers have found that *Lactobacillus* may improve balance of intestinal flora to improve feed utilization among chicks, thus reducing the amount of feed consumed while maintaining higher or equal production performance^18^. Furthermore, it was reported that *Firmicutes* were positively associated with fat storage and serum lipid levels. Thus, *Firmicutes* overload increased liver fatty acid synthesis and improved abdominal fat deposition^19^. It was recently discovered that amino acid transporters and ion transporters have been increasingly associated with bacterial groups in the intestinal flora of broilers^20^. Aviagen proposed that the disruption of normal bacterial flora balance in caeca may lead to metabolic disorders^21^. Some studies have shown that dietary supplementation with organic zinc can increase beneficial intestinal bacterial abundance and decrease harmful bacterial abundance^22–24^. Organic zinc was more than three times as efficient as inorganic zinc in improving growth and increasing resistance to *Edwardsiella ictalurid*^25^. This also corresponds to evidence illustrating that cecal microbes affect broiler lipid metabolism and thus growth performance.

The purpose of this study was to determine the optimal dose of MHA-Zn in LP diet and to investigate effect of different doses of MHA-Zn on production performance, meat quality and gut microbiota of broilers in 1.5% reduced protein diets.

## Results

### Growth performance

As shown in Table 1, the body weight gain in Group C was the highest among three low protein groups from the third week onwards and was no significant difference compared to Group A (*p* > 0.05). The feed intake and feed conversion ratio (FCR) of Group C were not significantly different from control group (Group A), except for the first week (*p* > 0.05). Among three LP diet groups, FCR of Group C was lower than that of Groups B and D besides the first two week (Table 1). Thus, the production performance of broilers fed LP diet with MHA-Zn (90 mg/kg) was the closest to those of normal protein (NP) group.

**Table 1:** Effects of different doses of MHA-Zn in low-protein diets on growth performance of broilers.

Item	A	B	C	D	SEM	*p*-value
Body weight gain (g)						
Week 1	163.01^a^	139.52^b^	136.01^b^	136.78^b^	3.548	< 0.01
Week 2	311.23^a^	262.52^b^	275.18^b^	261.99^b^	6.545	< 0.01
Week 3	465.94^a^	403.25^b^	446.32^a,b^	401.62^b^	11.550	0.099
Week 4	590.86^a^	468.22^c^	543.97^a,c^	440.74^b^	20.827	< 0.01
Week 5	641.20	615.13	667.01	628.28	21.760	0.892
Week 6	690.58	605.85	638.36	629.87	16.771	0.373
Feed intake (g)						
Week 1	181.28^a^	161.66^b^	160.81^b^	167.66^b^	2.842	< 0.01
Week 2	393.89	363.38	380.51	378.63	8.899	0.748
Week 3	709.15	623.88	651.56	593.87	22.209	0.613
Week 4	845.55	755.47	789.79	700.12	25.139	0.227
Week 5	996.03	1072.66	1098.88	1055.74	34.659	0.809
Week 6	1176.06	1150.40	1083.51	1101.97	25.899	0.625
FCR						
Week 1	1.11^b^	1.16^a,b^	1.18^a,b^	1.22^a^	0.016	0.059
Week 2	1.27	1.38	1.39	1.45	0.036	0.401
Week 3	1.52	1.53	1.47	1.48	0.047	0.971
Week 4	1.43^b^	1.61^a^	1.45^b^	1.58^a^	0.028	0.013
Week 5	1.56^b^	1.75^a^	1.65^a,b^	1.68^a,b^	0.031	0.149
Week 6	1.71^b^	1.90^a^	1.70^b^	1.75^a,b^	0.032	0.062

^a,b^Values in the same row with different superscripts are significantly different (*p* < 0.05). Group A, normal diet (110 mg/kg MHA-Zn); Group B, low-protein diet (70 mg/kg MHA-Zn); Group C, low-protein diet (90 mg/kg MHA-Zn); Group D, low-protein diet (110 mg/kg MHA-Zn). FCR, feed conversion ratio.

### Apparent total tract digestibility of nutrient

**Figure 1:**
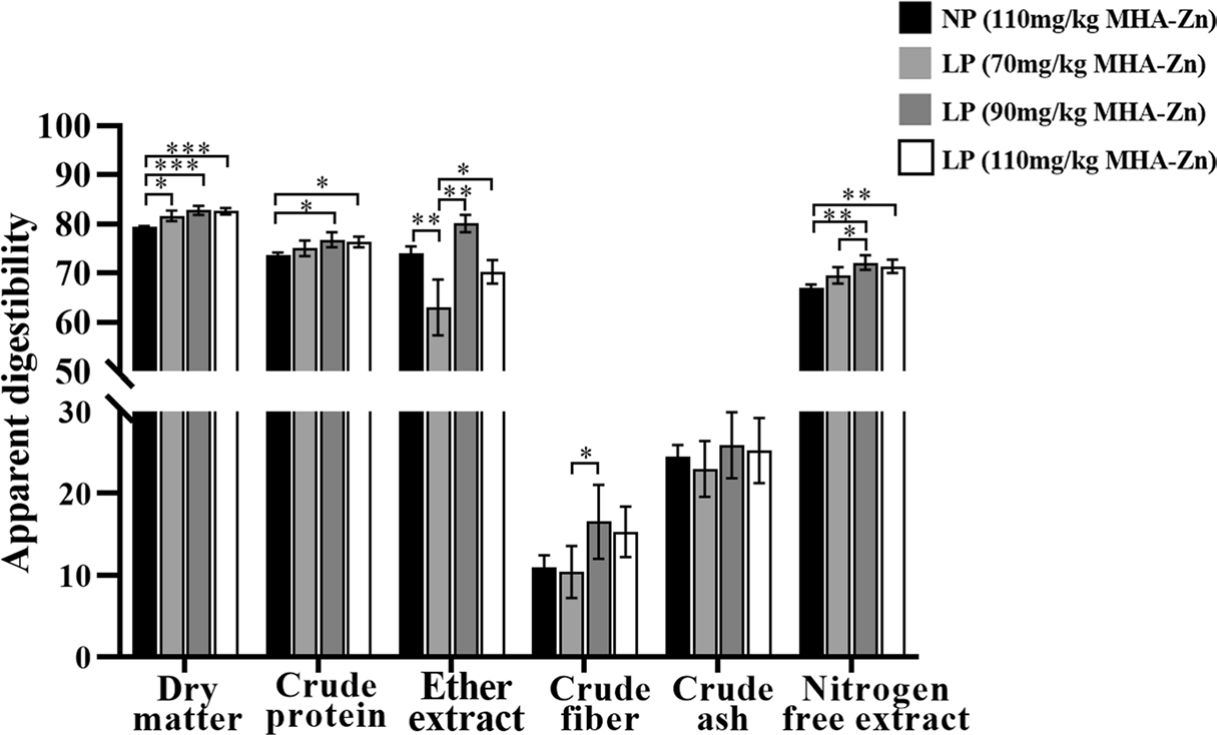
Apparent total tract digestibility of nutrients in broilers. The results are expressed as the means ± SDs, n = 3. *Indicates a difference at *p* < 0.05, **at *p* < 0.01, ***at *p* < 0.001.

As an important indicator, ATTD can reflect animal health and development. As shown in Fig. 1, the dry matter (DM) (*p* < 0.01), CP and nitrogen free extract (NFE) (*p* < 0.05) ATTD in Group C were markedly increased compared to that in Group A. The digestibility of DM was significantly higher in Group B than in Group A (*p* < 0.05). The apparent digestibility of CP was significantly higher in Group D than in Group A (*p* < 0.05).

### Slaughter performance

The results showed that there was no significant difference in the dressed yield or half-eviscerated yield among four groups at 42 days (*p* > 0.05), but the eviscerated yield of Group B was markedly decreased compared to that of Groups A and C (*p* < 0.01; *p* < 0.05). Moreover, the breast muscle yield was increased in NP diet group compared to LP diet groups (*p* < 0.01), but a trend in the opposite direction was seen between Group A and Group C in the leg muscle yield (*p* < 0.01). The edible parts were also compared statistically, and there was no difference between Group A and Group C in the sum of breast and leg muscle yield (edible parts), as shown in Table 2 (*p* > 0.05).

**Table 2:** Effects of different doses of MHA-Zn in low-protein diets on slaughter performance of broilers.

Item (%)	A	B	C	D	SEM	*p*-value
Dressed yield	92.11	91.42	91.42	91.44	0.173	0.475
Half-evisceration yield	84.11	83.23	84.00	83.63	0.186	0.342
Evisceration yield	71.51^a^	68.83^b^	71.18^a^	70.59^a,b^	0.371	0.037
Breast muscle yield	26.99^a^	20.58^c^	23.12^b^	22.99^b,c^	0.614	< 0.01
Leg muscle yield	21.01^b^	22.77^a,b^	24.09^a^	22.40^a,b^	0.391	0.035
Breast + Leg muscle yield	47.99^a^	43.36^b^	47.21^a^	45.39^a,b^	0.669	0.05

^a,b^Values in the same row with different superscripts are significantly different (*p* < 0.05). Group A, normal diet (110 mg/kg MHA-Zn); Group B, low-protein diet (70 mg/kg MHA-Zn); Group C, low-protein diet (90 mg/kg MHA-Zn); Group D, low-protein diet (110 mg/kg MHA-Zn).

### Organ index and meat quality

The results showed that there was no difference among four groups in the spleen index, bursa of Fabricius index or thymus index (*p* > 0.05) (Table 3). There was no significant difference in the meat quality among four groups, as shown in Table 4 (*p* > 0.05).

**Table 3:** Effects of different doses of MHA-Zn in low-protein diets on immune organ index of broilers.

Item (%)	A	B	C	D	SEM	*p*-value
Spleen index	0.11	0.12	0.14	0.12	0.006	0.447
Bursa of fabricius index	0.20	0.25	0.22	0.22	0.049	0.552
Thymus index	0.48	0.44	0.38	0.37	0.062	0.167

Group A, normal diet (110 mg/kg MHA-Zn); Group B, low-protein diet (70 mg/kg MHA-Zn); Group C, low-protein diet (90 mg/kg MHA-Zn); Group D, low-protein diet (110 mg/kg MHA-Zn).

**Table 4:** Effects of different doses of MHA-Zn in low-protein diets on meat quality of broilers.

Item	A	B	C	D	SEM	*p*-value
Meat color	75.60	76.25	78.20	75.39	0.926	0.725
pH	5.85	5.85	5.81	5.96	0.028	0.241
Water loss (%)	25.47	25.82	27.38	26.33	0.443	0.475
Cooking loss (%)	14.62	13.36	13.95	12.67	0.393	0.354

Group A, normal diet (110 mg/kg MHA-Zn); Group B, low-protein diet (70 mg/kg MHA-Zn); Group C, low-protein diet (90 mg/kg MHA-Zn); Group D, low-protein diet (110 mg/kg MHA-Zn).

### Serum chemistry analysis

**Figure 2:**
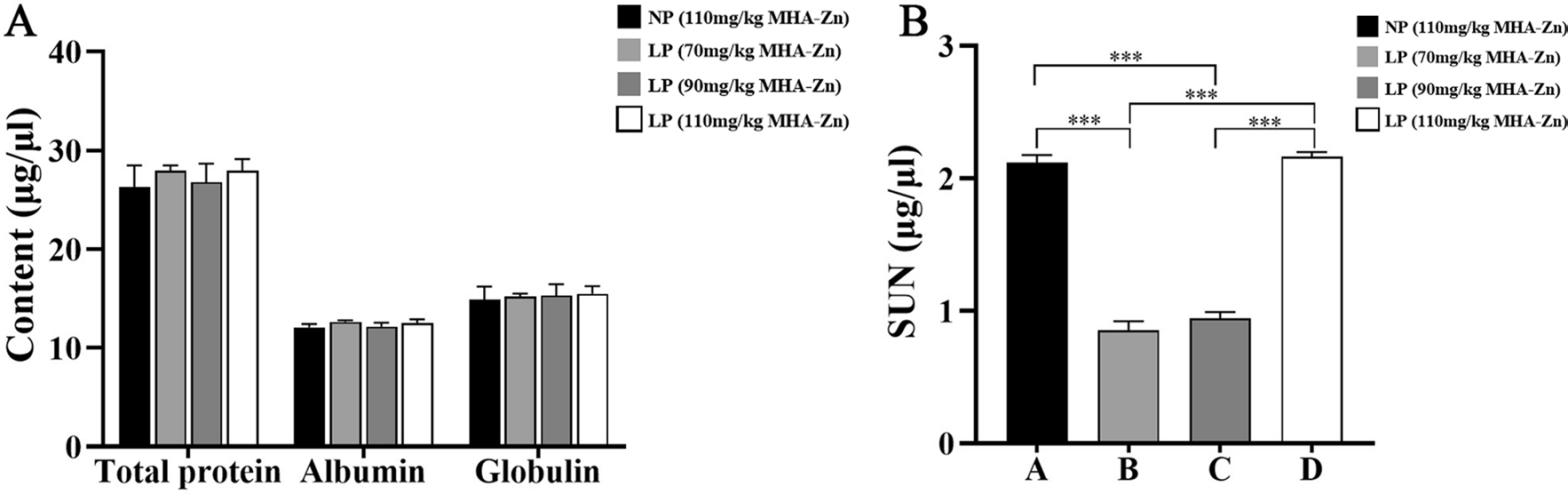
Serum biochemical parameters of broilers. (**A**) Total protein, albumin and globulin measurement in broilers. (**B**) SUN measurement in broilers. The results are expressed as the means ± SDs, n = 3. *Indicates a difference at *p* < 0.05, **at *p* < 0.01, ***at *p* < 0.001. SUN, serum urea nitrogen.

The level of serum biochemical parameters is an index that reflects degree of overall health and physical functioning of broilers. There were no significant differences in protein estimations (total protein, albumin and globulin) across groups (*p* > 0.05) (Fig. 2A). The results showed that SUN of Groups B and C was significantly lower than that of NP diet group (*p* < 0.01), similarly, of three LP diet groups (*p* < 0.01) (Fig. 2B).

### Ileal histomorphology

**Figure 3:**
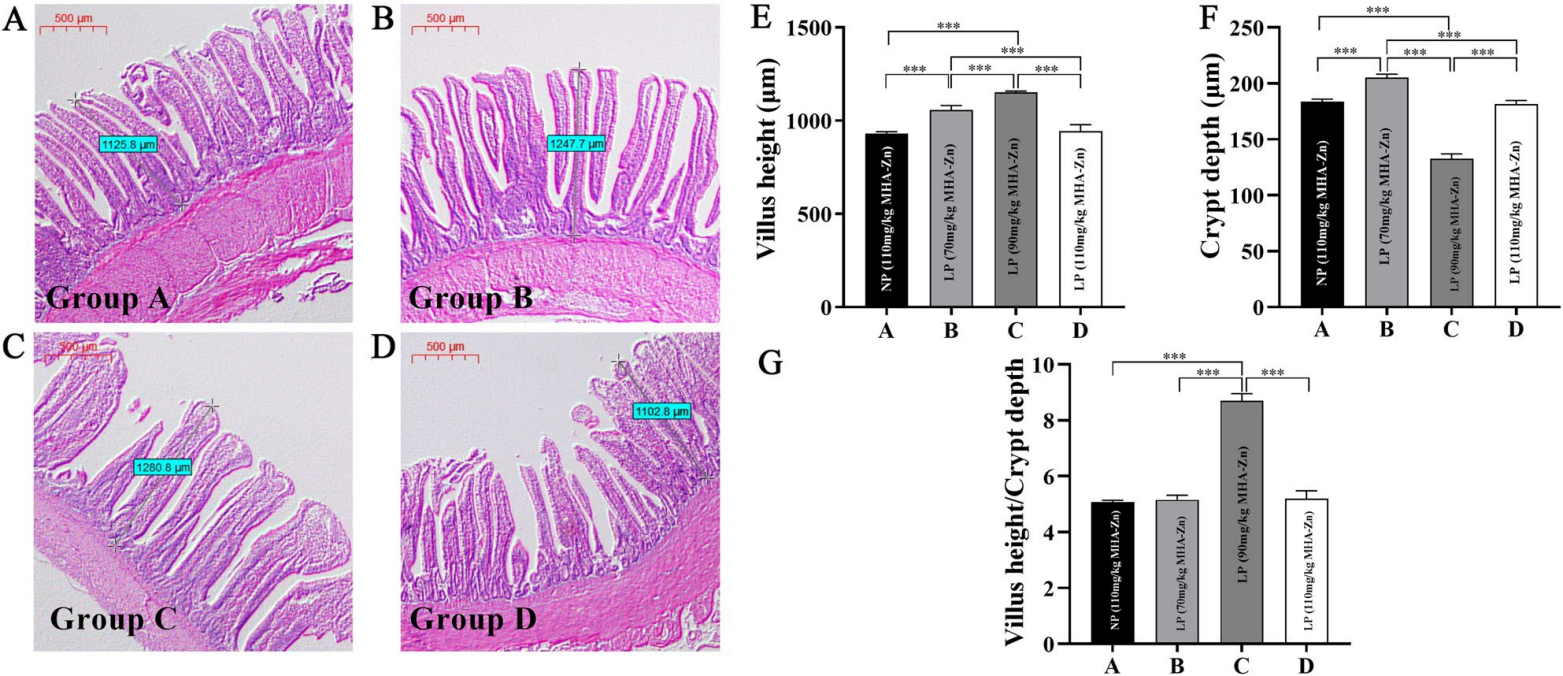
Ileal morphology of broilers. (**A–D**) Ileal morphological observation of broilers. (**A**) Group A, normal diet (110 mg/kg MHA-Zn); (**B**) Group B, low-protein diet (70 mg/kg MHA-Zn); **(C)** Group C, low-protein diet (90 mg/kg MHA-Zn); (**D**) Group D, low-protein diet (110 mg/kg MHA-Zn). The scale is 500 μm. (**E–G**) Ileal morphological analysis of broilers. (**E**) Villus height (μm). (**F**) Crypt depth (μm). (**G**) Villus height/crypt depth. The results are expressed as the means ± SDs, n = 3. *Indicates a difference at *p* < 0.05, **at *p* < 0.01, ***at *p* < 0.001.

Results showed that the height of villus in Group C was significantly greater than that in the other groups, and the depth of crypts determined by HE staining was significantly lower than that in the other groups (*p* < 0.01). The height of villus in Group B was significantly greater than that in Groups A and D (*p* < 0.01), and the same trend was observed for the depth of crypts (*p* < 0.01). Villus height/crypt depth (V/C) was also increased in intestine of broilers in treatment with 90 mg/kg MHA-Zn supplementation in LP diet groups (*p* < 0.01) (Fig. 3).

### Cecum microbiota and gene expression related to ileal absorption

The phenotypic data showed that including MHA-Zn (90 mg/kg) in low-protein diets improved broiler production performance, meat quality and ATTD. The potential mechanisms for this improvement remain unclear. Thus, cecum content samples and ileum tissue were collected from broilers fed low-protein diets with MHA-Zn (90 mg/kg) and normal diets (110 mg/kg MHA-Zn) to analyze cecum microbiota and expression level of genes related to ileal absorption.

### ASV analysis of cecum flora

**Figure 4:**
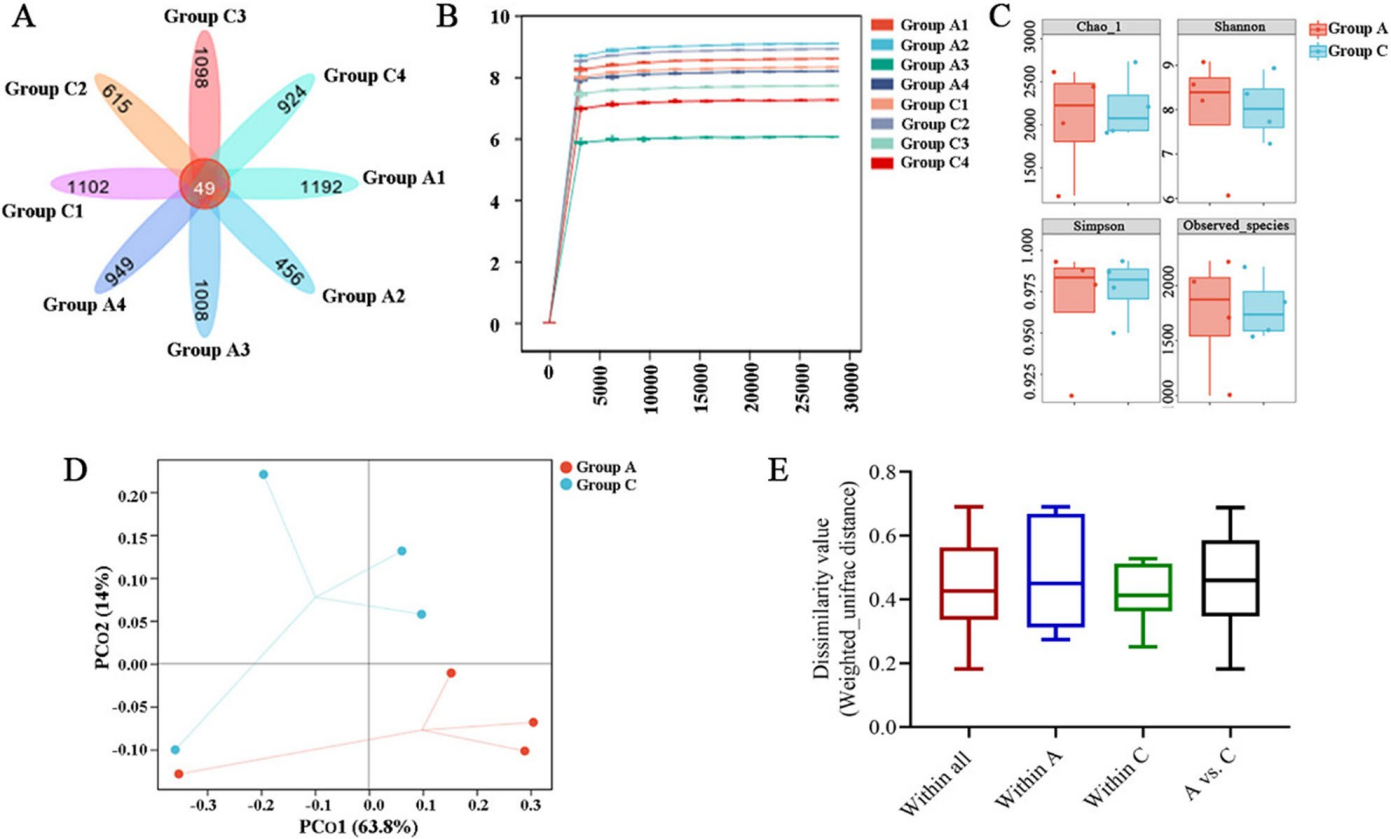
The diversity and structure of the cecal microbiota. (**A**) The number of ASVs that are unique to each group and shared between two groups. (**B**) Rarefaction curve. (**C**) Alpha diversity analysis. (**D**) Principal coordinate analysis (PCoA) plots based on weighted UniFrac distance analysis. (**E**) Sample distance figure based on weighted UniFrac analysis.

Sequence reads from the cecal metagenome were generated with high-throughput sequencing technology and subjected to quality control. A total of 510,450 sequences were generated from 8 cecal samples (4 samples from Groups A and C) after noisy sequences were discarded according to the minimum sequencing depth. As expected, the sequencing depth was 95% of the minimum sample sequence size in these samples (Table 5), thus the data were suitable for the following analysis. The number of valid sequences in all amplicon sequence variants (ASVs) was clustered with 100% agreement. Total 7344 ASVs were obtained from 8 samples, with 49 shared ASVs between two groups (Fig. 4A).

**Table 5:** Sequencing depth of cecal flora of broilers in control and experimental groups.

Sample	A	C
Sample1	63,456	67,879
Sample2	62,804	69,892
Sample3	59,247	62,236
Sample4	65,670	59,266

Group A, normal diet (110 mg/kg MHA-Zn); Group C, low-protein diet (90 mg/kg MHA-Zn).

### Alpha and beta diversity analysis

Rarefaction curves showed that sequencing depth was sufficient to cover biodiversity of samples, with curves approaching a plateau, and all samples reached desired level for this experiment (Fig. 4B). Alpha diversity analysis can indicate richness and diversity of species communities. The overall alpha diversity values (Chao1 index, Shannon, Simpson and Observed species) did not differ between two groups (Fig. 4C). Beta diversity analysis of broilers cecal microbiota compared composition of microbial communities among different samples. The principal coordinate analysis (PCoA) was calculated on weighted UniFrac distance, and findings indicated that bacterial communities in the cecal samples of Group A and Group C were not different (Fig. 4D,E).

### The structure of intestinal bacterial flora and gene expression related to ileal absorption

**Figure 5:**
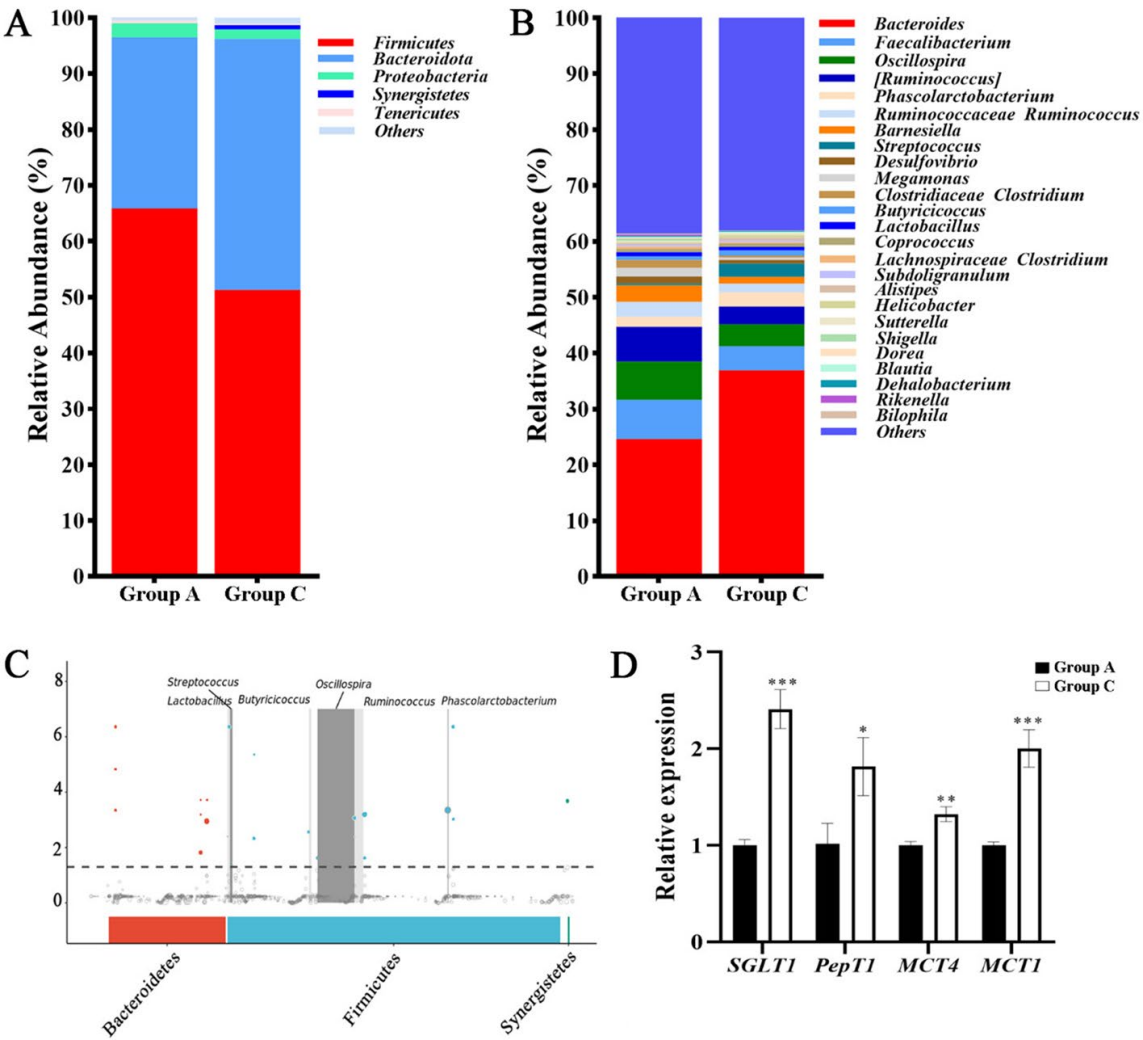
Differences in the cecal microbial composition and gene expression of broiler ilea between the two groups. (**A**) Bacterial community compositions at phylum level. (**B**) Bacterial community compositions at genus level. (**C**) Manhattan plots show cecum-enriched ASVs in Group C. All dots and circles in the figure occur at frequencies ≥ 0.3 in two groups; ASVs above the dashed line are significantly different between two groups. The solid dots represent ASVs that are significantly upregulated in Group C, while hollow circles represent ASVs that are significantly downregulated in Group C. The ASVs that are not significantly different between two groups are indicated below the dashed line (gray circles). The size of dot/circle represents abundance; different ASVs are colored by phylum level. (**D**) The gene expression of broiler ileal absorption. The results are expressed as the means ± SDs, n = 3. *Indicates a difference at *p* < 0.05, **at *p* < 0.01, ***at *p* < 0.001.

Considering changes in the structure of intestinal flora by diet, taxonomic compositions were analyzed for all groups at phylum and genus levels. The results from 16S rRNA gene library were displayed at the phylum level, showing a predominance of *Firmicutes* (65.88%), *Bacteroidetes* (30.62%), *Proteobacteria* (2.50%), *Tenericutes* (0.51%) and *Synergistetes* (0.028%) in Group A. In Group C, the five dominant phyla were *Firmicutes* (51.30%), *Bacteroidetes* (44.95%), *Proteobacteria* (1.67%), *Synergistetes* (0.75%) and *Tenericutes* (0.25%) (Fig. 5A).

At the genus level, the five most dominant genera with the highest relative abundances in Group A were *Bacteroides* (24.61%), *Faecalibacterium* (7.05%), *Oscillospira* (6.82%), *Ruminococcus* (6.19%), and *Phascolarctobacterium* (1.84%). In Group C, the five most dominant genera with the highest relative abundances were *Bacteroides* (36.91%), *Faecalibacterium* (4.30%), *Oscillospira* (3.93%), *Ruminococcus* (3.18%), and *Phascolarctobacterium* (2.56%) (Fig. 5B).

Interestingly, compared with Group A, the flora in the LP group (90 mg/kg MHA-Zn) significantly improved and included *Streptococcus*, *Lactobacillus*, *Butyricoccus*, *Oscillospira*, *Ruminococcus* and *Phascolarctobacterium* at ASV level (*p* < 0.01) (Fig. 5C). The expression levels of key genes related to ileal absorption are shown in Fig. 5D. The expression levels of ileal peptide transporter 1 (PepT1), sodium glucose cotransporter 1 (SGLT1), monocarboxylate transporter 4 (MCT4) and monocarboxylate transporter 1 (MCT1) were significantly increased in LP diet group (90 mg/kg MHA-Zn) compared with Group A (*p* < 0.05).

## Discussion

This study was conducted to investigate the impact of dietary supplementation with MHA-Zn at different concentrations in an LP diet on the growth-related performance and intestinal flora of broilers. It was reported in the literature that feeding poultry with reduced dietary CP content resulted in reduced performance and affected physiological markers^26,27^, but there were no significant ramifications for poultry health as long as the reduction in dietary CP content was less than 3%^8–10^. In this experiment, the CP level in treatment group was less than 1.5% lower than that in control group according to Technical Specification of Jilin Province Local Standard Broiler LP Diet (DB22/T 3207-2020). Additionally, a level of 110 mg/kg of Zn was provided in control group diet according to standardized management manuals of broilers^28^, which ensured that maximal performance was reached^11^. In the preliminary experiments, supplementation of MHA-Zn (90 mg/kg) in a low-protein diet significantly increased broilers growth performance. Except for the first two weeks, FCR in Group C (90 mg/kg MHA-Zn) was the closest to that in control group. This was consistent with the reports by Bueno et al.^29^ and Jahanian et al.^30^, who also suggested that supplementation with organic zinc (zinc acetate dihydrate, Zn-lysine chelate and MHA-Zn) significantly improved intake of feed and feed efficiency of broilers.

Considered an important performance indicator, ATTD is important for animal health and development. The higher digestion and absorption efficiency of feed, the more beneficial it is to animal growth^31^. When nutrient level of diet was changed, it had a significant impact on animals' ATTD. This study revealed that supplementation of broilers with MHA-Zn (90 mg/kg) in a low-protein diet significantly increased ATTD and improved feed utilization. This was consistent with the findings of Yang et al.^32^, who found that reducing dietary protein levels can improve apparent digestibility of DM and CP. It was believed that increasing CP digestibility improved protein deposition in broilers, thus increasing proportion of edible parts with higher protein content. Additionally, villus height and crypt depth are two common indices used to assess broilers intestinal integrity. Increased villus height indicates increased surface area for nutrient absorption, whereas increased crypt depth indicates rapid tissue turnover, which is typically associated with decreased nutrient digestion and absorption capacity^33^. In this study, the group provided MHA-Zn (90 mg/kg) had a significantly increased ileal villus height and decreased crypt depth in ileum compared with other groups. It was speculated that this may be one of the reasons for increased ATTD.

Meat quality is an important indicator for evaluating carcass yield and quality, which has significant implications for economic benefits of poultry industry. Poultry meat is an important animal product for human nutrition. The highest quality parts of broiler carcasses are breast and leg muscle, which contain high levels of protein, fat, and collagen^2^. Therefore, poultry breast and leg muscle yield (edible part) are the most indicative of meat quality. The results showed that dressed yield did not vary significantly among all groups (*p* > 0.05). These observations were consistent with the findings of others^29,34,35^. There were no significant differences in the sum of breast and leg muscle yield between broilers fed LP diet with MHA-Zn (90 mg/kg) and those fed NP diet, indicating that addition of 90 mg/kg MHA-Zn to LP diet did not reduce the proportion of edible part of broilers despite a reduction in feed intake. Thus, the addition of MHA-Zn (90 mg/kg) in LP diet could improve economic benefits of broilers production. Results in the study are consistent with findings of Zakaria et al.^36^ and Saenmahayak et al.^37^, and the level of Zn content had no significant effect on quality parameters of meat, such as pH, meat color, cooking loss and water holding capacity. Contrary results have been found in some studies^16,38^, it has been speculated that the causes for above differences may be age of broilers and rearing environments.

The immune organ index and serum biochemical indices are indicators of immune status and overall nutritional state of poultry. The results showed no significant differences in main evaluation indices in any group after measurement in the study, indicating that LP diet with MHA-Zn at doses employed did not cause immune system damage. This was in line with the findings of other studies^39^. Different findings were reported for other studies, which found that compared with inorganic Zn, zinc-methionine can significantly improve thymus index of poultry during feeding period^40^. This difference might have been caused by the use of different raw materials in feed formulation. It has been reported that SUN is a direct measure of total nitrogen excretion and is main nitrogen-containing substance resulting from protein decomposition^6,7^. The experimental results clearly showed that SUN levels in the MHA-Zn (70 mg/kg)-added group and MHA-Zn (90 mg/kg)-added group were significantly lower than those in other groups. These results revealed that an appropriate MHA-Zn content (≤ 90 mg/kg) was necessary to reduce NOx emissions.

The gut microbiome plays an important role in maintaining gut health, normal physiological functions, and productivity of poultry. This study was conducted to explore effects on cecal microbial communities when broilers were fed a normal diet and LP diet (90 mg/kg MHA-Zn). It has been previously shown that *Firmicutes* and *Bacteroidetes* phylum are two major phyla of mammalian gut bacteria^41^, which was consistent with the results of present study. Some reports have found that *Lactobacillus*, *Butyricoccus*, *Oscillospira*, *Ruminococcus* and *Phascolarctobacterium* are ubiquitous microflora present in intestinal tract of various animals. A high relative abundance of *Lactobacillus*, *Butyricoccus*, *Oscillospira* and *Phascolarctobacterium* has been observed in cecal microbiota of broilers. These bacteria can produce butyrate by fermenting CF, and *Butyricoccus* is among the greatest producers of short-chain fatty acid (SCFA) butyrate^42–44^. Butyrate can serve as an energy source to intestine epithelium, attenuate intestinal inflammation, improve intestinal barrier function and restrict propagation of pathogens^45^. These bacteria can also reduce the discharge of nitrogen emissions into environment as well as increase nutrient digestibility^46^. In addition, *Ruminococcus* is one of the oldest intestinal bacteria and plays an important role in metabolism; it obtains nutrients primarily by decomposing cellulose, a major component of CF in host digestive system^47^. These are considered to be factors in increased digestibility of CF. In present study, the addition of MHA-Zn (90 mg/kg) to LP diet significantly enriched the abundances of beneficial bacteria *Lactobacillus*, *Butyricoccus*, *Oscillospira*, *Ruminococcus* and *Phascolarctobacterium* in cecum of broilers compared with control group at ASV level. It is speculated that increased abundances of *Lactobacillus*, *Butyricoccus*, *Oscillospira*, *Ruminococcus* and *Phascolarctobacterium* in Group C may also be related to the increase in intestinal V/C, which promotes digestion and absorption of nutrients.

Many studies have delved into digestion and absorption genes to explore the mechanisms of digestion and absorption in animals. Research has shown that ileum is the main site of nutrient digestion and absorption in broilers^48^. Therefore, the experiment investigated expression of PepT1, SGLT1, MCT4 and MCT1 in broilers ileum. Since dietary proteins are absorbed as dipeptides and tripeptides rather than free amino acids in small intestine, PepT1, which is responsible for intestinal absorption of small peptides, is important nutritionally^49^. The sodium-glucose cotransporter SGLT1 is primarily responsible for glucose uptake^50^, and glucose is mainly a product of NFE. As a result, SGLT1 is closely related to NFE absorption. Monocarboxylate transporter MCT1 and MCT4 are H^+^-coupled transporters that mediate SCFA influx from lumen and efflux into blood^51^, while SCFAs are produced through CF fermentation. Moreover, MCT1 and MCT4 can improve absorption of small-molecule nutrients. They are beneficial for the absorption of nutrients. In this study, the expression of PepT1, SGLT1, MCT4 and MCT1 in broilers ileum was significantly increased in MHA-Zn (90 mg/kg)-added group (*p* < 0.05). It is supposed that this may be the reason for higher DM, CP and NFE digestibility in Group C than in control group. These results were consistent with those of previous studies showing that upregulation of expression of PepT1, SGLT1, MCT4 and MCT1 improved nutrient digestibility^49,52–54^.

## Conclusions

These results suggest that the addition of MHA-Zn (90 mg/kg) improved production performance and meat quality of broilers fed an LP diet, reaching a level close to that of broilers fed an NP diet while reducing feed costs and nitrogen excretion. The addition of 90 mg/kg MHA-Zn to the LP diet improved apparent nutrient digestibility, intestinal histomorphology, beneficial flora of cecum and gene expression related to ileal absorption.

## Materials and methods

### Experimental design and diets

Broilers were provided by Jilin Dexiang Animal Husbandry Co., Ltd. All experimental protocols were approved by the College of Animal Science of Jilin University Ethics Committee (SY202107101). All methods were performed in accordance with the relevant guidelines and regulations. All methods used in this study are reported in accordance with ARRIVE guidelines.

A total of 216 one-day-old mixed-sex Arbor Acres (AA) broilers were randomly allotted to 4 groups consisting of 3 replicates with 18 broilers per replicate, and the groups were carried out until day 42. There was no significant difference in starting weight among these groups. All broilers were raised flat on iron nets in the same environment. Broilers were given access to feed and water ad libitum and provided routine immunization^55^. The temperature in the chicken house was kept at 33 °C for first week and then gradually reduced by 3 °C per week to a final temperature of approximately 26 °C, and ambient humidity was controlled at 50–70%. Throughout the experiment, broilers were exposed to light for 23 h per day.

The experimental broilers were grouped as shown in Table 6. The broilers in control group were fed a basic diet, whereas those in test groups were fed diets in which CP was reduced by 1.5% following the Technical Specification of Jilin Province Local Standard Broiler LP Diet (DB22/T 3207-2020). Novus International Trading Company provided the MHA-Zn that was added to the diet (Shanghai, China). The basal diet was formulated according to NRC (1994) recommendations (Table 7).

**Table 6:** Experimental design.

Item	Group	CP level	MHA-Zn level
Control	A	NP	110 mg/kg
Treatment	B	LP	70 mg/kg
	C	LP	90 mg/kg
	D	LP	110 mg/kg

CP, crude protein; NP, normal protein; LP, low protein; MHA-Zn, methionine hydroxyl analogue chelated zinc.

**Table 7:** Ingredient and nutrient composition of normal and low-protein basal diet.

Ingredient (%)	0–14 days		15–28 days		29–42 days	
	NP	LP	NP	LP	NP	LP
Corn—fine	55.49	61.31	61.96	69.49	65.98	73.18
Soybean meal	35.60	31.70	29.10	23.50	25.40	20.10
Fat—soybean oil	2.50	0.80	3.10	1.10	3.42	1.50
Corn gluten meal	2.50	2.50	2.50	2.50	2.50	2.40
DCP	1.55	1.59	1.57	1.66	1.25	1.34
Calcium carbonate 38%	1.06	0.98	0.55	0.54	0.42	0.41
NaCl	0.38	0.28	0.31	0.30	0.21	0.21
DL-methionine	0.27	0.21	0.23	0.19	0.19	0.18
Sodium bicarbonate 24.1%	0.21	0.21	0.21	0.21	0.20	0.20
l-Lysine HCl 78%	0.12	0.10	0.14	0.17	0.14	0.18
Trace element premix	0.10	0.10	0.10	0.10	0.10	0.10
Choline chloride—50	0.10	0.10	0.10	0.10	0.10	0.10
Vitamin premix	0.05	0.05	0.05	0.05	0.05	0.05
Salinomycin 12%	0.05	0.05	0.05	0.05		
Thermostable phytase20000	0.01	0.01	0.01	0.01	0.01	0.01
l-Threonine 98.5%			0.01	0.02	0.02	0.03
Compound enzyme preparation	0.01	0.01	0.01	0.01	0.01	0.01
Total	100.00	100.00	100.00	100.00	100.00	100.00
Nutrition level analysis						
ME (MJ/kg)	11.88	11.67	12.25	12.01	12.54	12.31
Crude protein (%)	22.40	20.98	19.90	17.90	18.50	16.58
Ether extract (%)	4.96	3.43	5.69	3.89	6.10	4.36
Crude fiber (%)	2.36	2.32	2.24	2.16	2.17	2.10
Lysine (%)	1.34	1.22	1.18	1.06	1.08	0.97
Methionine (%)	0.62	0.54	0.55	0.48	0.49	0.45
Calcium (%)	0.85	0.82	0.65	0.65	0.52	0.52
Total phosphorus (%)	0.64	0.64	0.62	0.61	0.55	0.55
Zn (mg/kg)^a^	26.02	25.33	23.90	23.31	23.08	22.01

The mineral premix provided per kilogram of diet was as follows: Cu (as copper sulfate), 15 mg; Fe (as ferrous sulfate), 52 mg; Mn (as manganese sulfate), 115.2 mg; I (as potassium iodide), 1.14 mg; Se (as sodium selenite), 0.30 mg. The vitamin premix provided per kilogram of diet was as follows: vitamin A, 50,000 IU; vitamin D_3_, 12,500 IU; vitamin E, 90 IU; vitamin K_3_, 15,000 mg; vitamin B_1_, 10,000 mg; vitamin B_2_, 35,000 mg; vitamin B_6_, 15,000 mg; vitamin B_12_, 100 mg; vitamin B_3_, 150,000 mg; vitamin B_5_, 50,000 mg; vitamin B_9_, 7,000 mg; and vitamin B_7_, 350 mg. NP, normal protein; LP, low protein. ^a^The Zn content was measured using ICP‒OES.

### Sample collection and preparation

At day 39, one broiler was selected from each replicate for single-cage rearing to collect excreta for a four-day period to evaluate ATTD. The fresh excreta per cage was weighed once a day and frozen at − 20 °C for further analysis. At day 42, six broilers whose weights were close to average weight of the group were selected randomly from each treatment group (two broilers per replicate pen). Then, all 24 broilers were weighed and sacrificed by exsanguination, and 5 mL of blood was collected in a centrifuge tube. All immune organs (thymus, spleen, and bursa of Fabricius) were collected from the broilers and immediately weighed. Approximately 3 cm of the ileum was isolated and washed with saline solution (0.9% NaCl). For morphological measurements, the middle 2 cm of ileum was collected intact and fixed in 4% paraformaldehyde. Samples (0.5 cm) were collected from both ends of the central slice, placed in a centrifuge tube with RNAstore (Trans, Beijing, China), and stored at − 80 °C for qRT‒PCR analysis. The contents of each broiler cecum were aseptically collected in frozen storage tubes, quickly packed in dry ice, and sent out for analysis of the composition of gut bacterial communities. At the end of experiment, slaughter performance was measured and calculated according to poultry production performance terms and metric statistics method.

### Measurements of growth performance

The body weight and feed intake of broilers were recorded once a week during experiment. On day 42, the weekly FCR was calculated.

### Apparent total tract digestibility of nutrient

Four days of excreta and feed intake per cage of broilers were recorded and pooled in replicates for further analysis. They were analyzed for DM, CP, EE, CF, crude ash, and NFE according to AOAC procedures and as described by previous studies^56,57^.

$${\text{ATTD}}\;(\% ) = 100 \times ({\text{Nutrient}}\;{\text{in}}\;{\text{feed}} - {\text{Nutrient}}\;{\text{in}}\;{\text{excreta}})/{\text{Nutrient}}\;{\text{in}}\;{\text{feed}}$$

### Measurements of slaughter performance

Slaughter performance, including dressed yield (%), half-eviscerated yield (%), eviscerated yield (%), breast muscle yield (%), leg muscle yield (%), and sum of breast and leg muscle yield (%), was measured and calculated according to the poultry production performance terms and metric statistics method^58^. Dressed weight is the weight of poultry after bloodletting, removal of feathers, foot cuticle, toe shell and beak shell. Half-eviscerated weight is the weight of a carcass minus its trachea, esophagus, crop, intestine, spleen, pancreas, gall bladder, reproductive organs, stomach contents, and keratin. Eviscerated weight is taken as half-eviscerated weight minus the weight of heart, liver, muscle stomach, glandular stomach, abdominal fat, head, and foot.

$${\text{Dressed}}\;{\text{yield}}\;(\% ) = 100 \times \frac{{{\text{dressed}}\;{\text{weight}}\;({\text{g}})}}{{{\text{body}}\;{\text{weight}}\;({\text{g}})}}$$

$${\text{Half - eviscerated}}\;{\text{yield}}\;(\% ) = 100 \times \frac{{{\text{half - eviscerated}}\;{\text{weight}}\;({\text{g}})}}{{{\text{body}}\;{\text{weight}}\;({\text{g}})}}$$

$${\text{Eviscerated}}\;{\text{yield}}\;(\% ) = 100 \times \frac{{{\text{eviscerated}}\;{\text{weight}}\;({\text{g}})}}{{{\text{body}}\;{\text{weight}}\;({\text{g}})}}$$

$${\text{Breast}}\;{\text{muscle}}\;{\text{yield}}\;(\% ) = 100 \times \frac{{{\text{breast}}\;{\text{muscle}}\;{\text{weight}}\;({\text{g}})}}{{{\text{eviscerated}}\;{\text{weight}}\;({\text{g}})}}$$

$${\text{Leg}}\;{\text{muscle}}\;{\text{yield}}\;(\% ) = 100 \times \frac{{{\text{leg}}\;{\text{muscle}}\;{\text{weight}}\;({\text{g}})}}{{{\text{eviscerated}}\;{\text{weight}}\;({\text{g}})}}$$

### Determination of organ index

The spleen index, bursa of Fabricius index and thymus index of broilers were calculated as follows^59^:

$${\text{Organ}}\;{\text{Index}}\;(\% ) = 100 \times \frac{{{\text{Organ}}\;{\text{weight}}\;({\text{g}})}}{{{\text{body}}\;{\text{weight}}\;({\text{g}})}}$$

### Measurements of meat quality

Broilers were fed experimental diets until 42 days, after which left breast muscle was collected for the meat quality analysis. Meat quality was evaluated based on pH, meat color, cooking loss and water loss. The specific operations are as follows: the meat color and pH of breast muscle samples were assessed at three different sites using a carcass color tester (OPTO-STAR, Beijing Bulader Technology Development Co., Ltd., Denmark, Germany) and a portable pH meter (pH-STAR, Beijing Bulader Technology Development Co., Ltd., Denmark, Germany). The pH meter was calibrated using standard buffer solutions of pH 4.0 and 7.0. Cooking loss and water loss were measured as described by Ni et al.^60^. Approximately 1 g (W1) breast muscle samples were packaged in cooking bags and placed in a water bath at 80 °C until the central temperature reached 70 °C. After cooling, the samples were weighed again (W2) to calculate the cooking loss as follows: Cooking loss (%) = (W1 − W2)/W1 × 100%. Water loss was analyzed with a digital dilatometer (C-LM3B, Tenovo, Beijing, China). The breast muscle samples of about 1 g (W1) were weighed and 10 layers of filter paper were placed on the top and bottom of the sample. The covered sample was then placed on the dilatometer platform for 5 min at a pressure of 68.66 kPa and the weight of the muscle sample (W2) was measured again to calculate the calculated amount of water released as follows: Water loss (%) = (W1 − W2)/W1 × 100%.

### Determination of serum physiological parameters

After allowing the blood to stand at room temperature until completely coagulated, it was centrifuged at 4000 r/min for 15 min at 4 °C. The serum was then pipetted using a micropipette into 1.5 mL centrifuge tubes and stored at − 20 °C. The serum biochemical indices were measured using commercial kits (Meikang Biotechnology, Ningbo, China) and an automatic biochemical analyzer (MS-880B, Medicalsystem Biotechnology Co., Ltd, China), and the indices measured included serum total protein (μg/μL), albumin (μg/μL), globulin (μg/μL), and urea nitrogen content (μg/μL).

### Determination of ileal histomorphology

The broiler ileal tissues were fixed with 4% paraformaldehyde, embedded in paraffin and cut into 5 μm sections. Tissues were stained with hematoxylin and eosin (HE) after deparaffinization (Changchun Xavier Biotechnology Co. Changchun, China). Under a light microscope (X 40 magnification) with the Slide Viewer (version 2.5.0; 3DHISTECH Ltd., Budapest, Hungary) image-analyzing system, villus height (μm) and crypt depth (μm) were measured, and V/C was calculated^61^.

### DNA extraction and cecal microbiota analysis

On day 42, microbial DNA was extracted from cecum content samples using the OMEGA Soil DNA Kit (M5635-02) (Omega Bio-Tek, Norcross, GA, USA), following manufacturer’s instructions, and stored at − 20 °C prior to further analysis. The quantity of extracted DNA was measured using a Nanodrop NC2000 spectrophotometer (Thermo Fisher Scientific, Waltham, MA, USA) and agarose gel electrophoresis. PCR amplification of bacterial 16S rRNA gene V3–V4 region was performed using the forward primer 338F (5′-ACTCCTACGGGAGGCAGCA-3′) and the reverse primer 806R (5′-GGACTACHVGGGTWTCTAAT-3′). Sample-specific 7-bp barcodes were incorporated into the primers for multiplex sequencing. The Quant-iT PicoGreen dsDNA Assay Kit (Invitrogen, Carlsbad, CA, USA) was used to quantify PCR amplicons after they had been purified using Vazyme VAHTSTM DNA Clean Beads (Vazyme, Nanjing, China). After individual quantification step, amplicons were pooled in equal amounts, and pair-end 2 × 250 bp sequencing was performed using the Illumina MiSeq platform with MiSeq Reagent Kit v3 at Shanghai Personal Biotechnology Co., Ltd. (Shanghai, China). Sequencing and bioinformatics were performed on QIIME2 platform (version 2019.4)^62^. The sequencing results were analyzed based on ASVs^63^. Briefly, raw sequence data were demultiplexed using the demux plugin followed by primer cutting with cutadapt plugin^64^. Sequences were then quality filtered, denoised, and merged and chimera were removed using the DADA2 plugin^65^. Sequence data analyses were mainly performed using QIIME2 and R packages (v3.2.0). ASV-level alpha diversity indices, such as Chao1 richness estimator, Observed species index, Shannon diversity index, and Simpson index, were calculated using the ASV table in QIIME2 and visualized as box plots. Beta diversity analysis was performed based on weighted UniFrac distance matrices and displayed by PCoA. Taxa abundances at the ASV level were statistically compared among samples or groups and visualized as Manhattan plots using an R script and metagenomeSeq package (based on frequency of ASV in two groups ≥ 0.3).

### RNA extraction and real-time quantitative polymerase chain reaction

The mRNA expression levels of PepT1, SGLT1, MCT4 and MCT1 genes in ileal tissue were quantified by real-time quantitative PCR. β-Actin was used as a reference gene for normalization. Additional information on the primers used in this study is shown in Table 8^66–68^. The total RNA of ileum tissue samples was extracted with FastPure Cell/Tissue Total RNA Isolation Kit according to manufacturer’s instructions (Vazyme, Nanjing, China), and the integrity of RNA was assessed by visualization on agarose gel. The A260/A280 nm values of the RNA samples were within the acceptable range of 1.8–2.1. A reverse transcription kit (Trans, Beijing, China) was used to generate cDNA, and a SYBR Green Mix Kit was used for quantitative PCR (Trans, Beijing, China). The 48-well plate was then placed in a real-time fluorescent quantitative PCR instrument and melted for 30 s at 95 °C, followed by thermal cycling at 95 °C for 5 s, 60 °C (annealing temperature) for 30 s, and 72 °C for a 30 s extension. The number of cycles was generally 40, and it could be adjusted depending on primer reactions. The 2^−ΔΔCt^ method was used to calculate relative mRNA levels of genes from fluorescent quantitative results.

**Table 8:** Primer sequences used for qRT‒PCR.

Gene	Primer nucleotide sequence (5′ → 3′)	Product length (bp)	Reference
PepT1	F: TTCCCATGGAGTCAACAGGC R: GGCTGCTGCATTCTTGATGG	146	Yu et al.^66^
SGLT1	F: TGTGGGCATAGCAGGAACAG R: TACTCCGGCATTGTCACCAC	141	Yu et al.^66^
MCT4	F: GCTGGTCTCAAGTGGGTTAG R: CCACCGTAATCGACAGACATAG	107	Zhao et al.^67^
MCT1	F: GTGACCATTGTGGAGTGCTG R: CCACAGGCCCAGTATGTGTA	101	Qu et al.^68^
β-Actin	F: ACCGGACTGTTACCAACACC R: CCTGAGTCAAGCGCCAAAAG	116	Yu et al.^66^

F, forward; R, reverse; PepT1, peptide transporter 1; SGLT1, sodium glucose cotransporter 1; MCT4, monocarboxylate transporter 4; MCT1, monocarboxylate transporter 1.

### Data analysis

Statistical analyses were performed by one-way analysis of variance, and gene expression data were analyzed by t test using SPSS 26.0 software (SPSS, Inc., Chicago, IL, USA). Histograms were made using GraphPad Prism 8 software (San Diego, CA, USA). QIIME2 and R (v3.2.0) were primarily used to analyze gut microbiota sequence data. The results are presented as the mean ± SD. Significant differences at *p* < 0.05, 0.01 and 0.001 are indicated as *, ** and ***, respectively.

## Data Availability

The datasets generated and/or analyzed during the current study are available in the NCBI sequence read archive under the Accession Number PRJNA953756.
